# Radiocarpal Fracture Dislocation: Minimally Invasive Treatment Assisted by Arthoscopic – Case Report

**DOI:** 10.1055/s-0044-1790593

**Published:** 2024-12-27

**Authors:** Marcio Aurelio Aita, Ricardo Kaempf de Oliveira, Bruno Gianordoli Biondi, Gustavo Luis Rodriguez Gómez, Marcos Jun Tamura, Gustavo Mantovani Ruggiero

**Affiliations:** 1 Departamento de Cirurgia, Ortopedia e Trauma, Divisão de Mão e Microcirurgia, Faculdade de Medicina do ABC, São Bernardo do Campo, SP, Brasil; 2 Departamento de Ortopedia e Trauma, Santa Casa de Porto Alegre, Porto Alegre, RS, Brasil; 3 Departamento de Ortopedia e Trauma, Divisão de Mão e Microcirurgia, Faculdade de Medicina do ABC, Santo André, SP, Brasil; 4 Departamento de Cirurgia de Mão, Clínica de la Mano de Buenos Aires, Buenos Aires, Argentina; 5 Departamento de Ortopedia e Trauma, Hospital Assunção, Rede D'or, São Bernardo do Campo, SP, Brasil; 6 Departamento de Cirurgia Plástica, Universita Degli Studi Di Milano, Milão, Itália

**Keywords:** carpal bone, joint dislocations, radius fractures, wrist injuries

## Abstract

To measure the life quality, clinical-functional outcomes of a patient who had undergone acute reconstruction of radio scapho capitate (RSC), radio lunate (RLL) ligaments, using brachiorradialis tendon in treatment of radiocarpal fracture dislocation. 21-years-old, man with radiocarpal fracture dislocation in his left wrist, after motorcycle accident. Percutaneous screw fixation of the distal radius and acute reconstruction of the RSC and RLL was performed, assisted by arthroscopy. One year later, the patient experienced good evaluation. ROM was full, grip strength was 96% compared with the unaffected side were obtained. Wrist radiographic aspects showed fracture healed and radiocarpal joint congruency. Good stability and joint congruency of the radiocarpal joint were obtained and improving the life quality of that patient. Radiocarpal fracture dislocation management is difficult and complicated. There is no consensus. As there is still a lack of long-term results, the indications for surgery, and options, type of the intervention have been a matter of controversy in the literature. Would radiocarpal (RC) joint be stable when reconstruction of the radiocarpal ligaments, using brachiorradialis tendon was obtained? Is it possible to reduce and to maintain stable radiocarpal joint with this technique? The clinical relevance of this work is our suggestion of reconstruction of the RC ligament to improve this treatment. We believe that this will maintain a stable and functional wrist. We agree that the best time to perform corrections is as soon as possible and we prefer to reconstruct the RC ligament with suture or temporary or permanent radioscapholunate arthrodesis.

## Introduction

Radiocarpal fracture-dislocation is an uncommon traumatic disorder associated with injuries to the radiocarpal (RC) and radioulnar ligaments (RU). Diagnosis is delayed because of the lack of radiographic findings, and is made following chronic failure (instability) of the joint and wrist pain. Treatment of acute dislocation usually involves stabilization and ligament suture. In this patient, we stabilized the radiocarpal joint by RC reconstruction using a brachiorradialis tendon graft.

## Case Description



**Video 1** Clinical case: pre-, intra-, and postoperative aspects: from diagnosis to daily activities return.


**Fig. 1:**
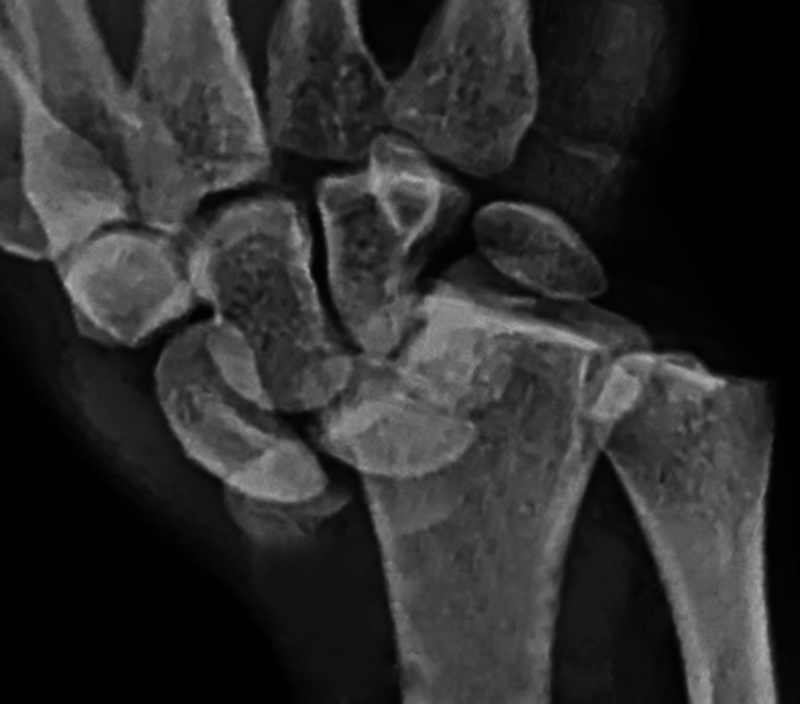
Wrist radiographic preoperative aspect – radiocarpal fractures dislocation.

A 21-year-old patient with motorcycle injury (politrauma) was presented with pain and deformity on the left dominant forearm. Initial radiographs revealed Radio carpal and DRUJ incongruence with radial styloid fracture ( Fig. 1 ). The patient had received treatment with closed reduction and percutaneous fixation (Headless compression screw HCS®, Synthes®, Davos, Switzerland) in styloid radial. After, physical examination revealed RC/DRUJ unstables, and the ulnar head had dorsal prominence. We decided (intra-operative) to perform wrist arthroscopy and diagnosed complex lesion: radio carpal ligaments and TFCC foveal avulsion (hook and trampolim tests were positive). Therefore, addressed the radio carpal instability by reconstruction of the radio carpal ligaments (RSC,RLL) using BR tendon.

The patient was kept with a long removable splint above the elbow for two weeks after the surgery, with his wrist in a neutral position for the improvement of the natural regain of pronation and supination. We started occupational therapy for the improvement of the forearm range in the first week after surgery. Four months after, the patient achieved with no pain and a stable radio carpal joint and DRUJ, exhibited good range of motion (ROM) for wrist, forearm, elbow and all digits. DASH score of 6, a VAS score of 0, and grip strength of 96% as compared with the opposite wrist. X-ray images revealed the articular congruency of DRUJ, styloid radial healing, and better bone attachment to the implants ( Video 1 ).

## Surgical Technique

**Fig. 2:**
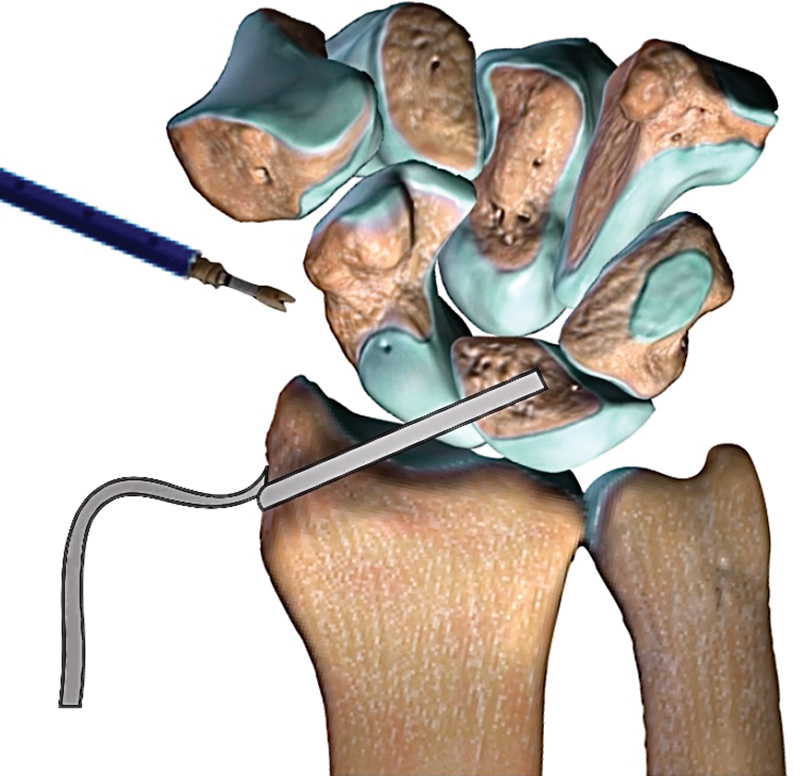
Schematic draw: reconstruction of radiolunate ligament.

**Fig. 3:**
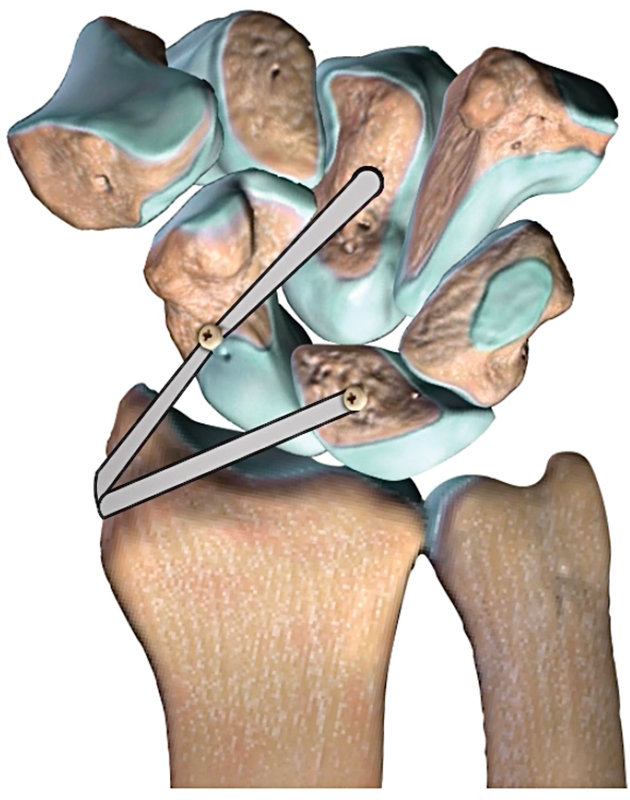
Schematic draw: reconstruction of radio scaphocapitate ligament.

**Fig. 4:**
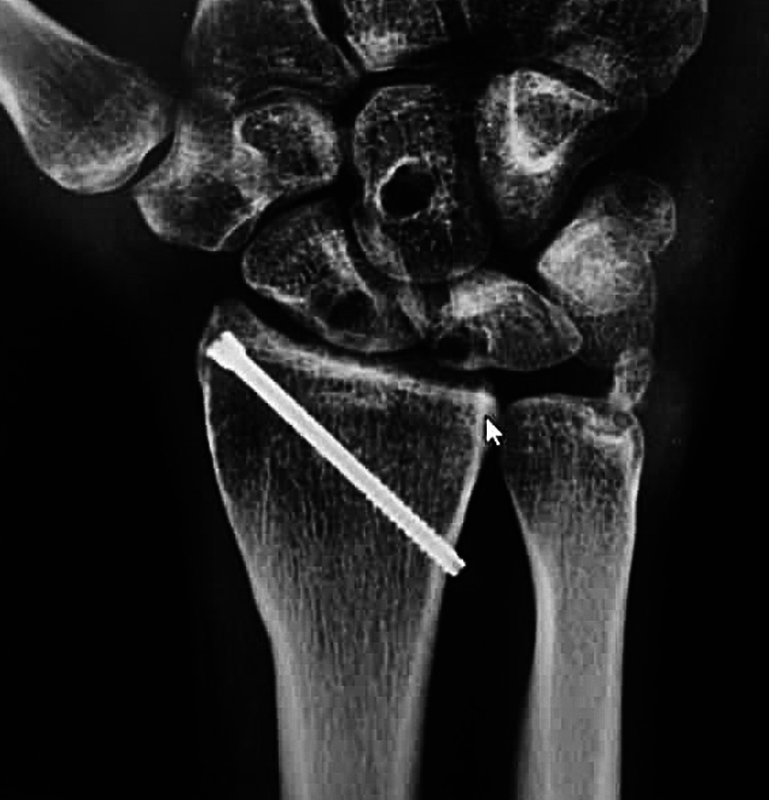
Postoperative aspects of anteroposterior wrist radiography.

**Fig. 5:**
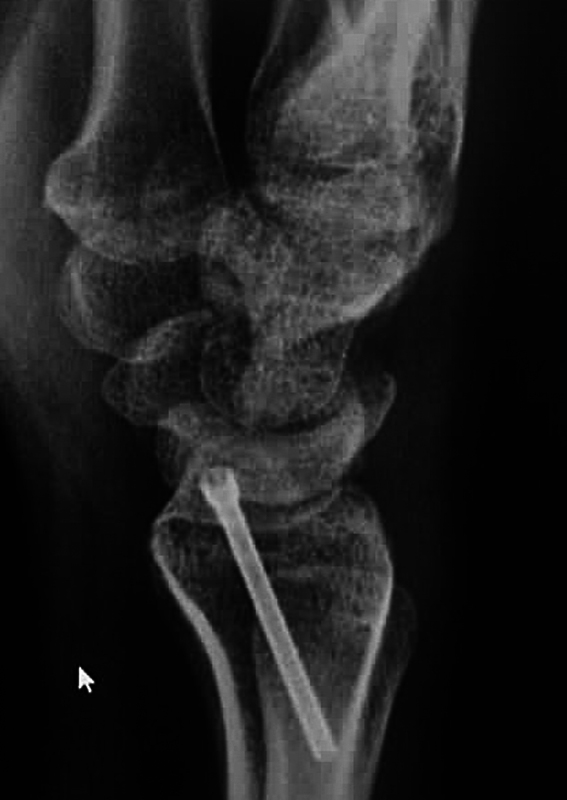
Postoperative aspects of wrist radiography in lateral view.

Wrist radial approach, wherein the BR tendon was identified, harvested, and shared in two parts: to RSC and other part to RLL. Therefore, we prepared with an internal brace (FiberTape® wire suture, Arthrex Inc., Naples, FL) by sectioning the tendon on its muscle transition and preserving the insertion on the radius styloid. After, we employed a medial ulnar approach for the reinsertion of TFCC in ulna by using the DX® anchor, through transverse tunnel, according the technique recommended by the manufacturer. After, we created three tunnels (scaphoid, lunate and capitate) by using a 3.5-mm cannulated drill, according the technique described by the authors, assisted by arthroscopy. We passed the BR tendon graft through the tunnel and kept it tensed on the palmar face on the lunate first ( Fig. 2 ) and, scaphoid and capitate ( Fig. 3 ) after, thereby providing stability between the radiocarpal joint. The definitive implants on the scaphoid, capitate and lunate were only inserted after accomplishing stability (DX®). ( Figs. 4 , 5 and Video 1 ).

## Discussion

Conventional treatment suggests open reduction and fixation of the styloid radial (screw, k-wires or specific plate) and direct suture repair the radio carpal and TFCC ligaments with k-wires. 1

In acute lesions, it is possible directly repair the radio carpal ligaments and TFCC, however, there is no consensus to the overall management.

In chronic lesions (the most common), we often need to reduce RC / DRUJ. Moreover, there is a need for procedures such as: Wrist partial arthrodesis, shortening the ulna and reconstructing the radio carpal ligaments. Aita et al. 2 published reconstruction of the RSC and obtained promising results for the stabilization of wrist and prevention of the osteoarthritis.

Aita and Mantovani 3 suggested algorithm for treating ligament lesions and introduced the wrist arthroscopy and internal brace® for the repair/reconstruction of intrinsic/extrinsic wrist carpal ligaments. It was advantageous as a direct view of the articular structures, avoid wrist dorsal capsule incision, to preserve upper limb proprioception, thereby avoiding other surgical sites.

Potter et al. 4 described the “spanning” as a stabilizer of the radio carpal joint, as opposed direction to that treat here, using the specific radio carpal plate with temporary arthrodesis and achieved positive results.

Here, we suggest that “internal brace” is also sufficient to treat RC and DRUJ traumatic instabilities, as this is an anatomical repair/reconstruction similar to that of the RSC/RLL or TFCC reconstruction. All procedures preserve the stability and mobility of the wrist and to increase the strength of the reconstruction and its protection. 3

The authors have a wide range of experience of using the BR tendon graft, offered a promising and efficient solution on this case and appears to be reproducible for future surgical interventions. 5

We agree that a long-term result, especially on a young patient as this one, is uncertain, and there will be some known and unknown complications in the future. There might be an additional need of another salvage procedure; however, the excellent functional outcome on this mid-term follow-up seems to justify this indication on these special circumstances.
