# Supplementary material for: Radiocarpal Fracture Dislocation: Minimally Invasive Treatment Assisted by Arthoscopic – Case Report
Source: Rev Bras Ortop (Sao Paulo). 2024 Dec 27;59(Suppl 2):e168–72. doi: 10.1055/s-0044-1790593 (PMC11679616; doi:10.1055/s-0044-1790593)
Supplement: Related texts

# Research article: Fratura-luxação radiocárpica: Tratamento minimamente invasivo assistido por artroscopia – Relato de caso

**Keywords:** fraturas do rádio, luxações articulares, ossos do carpo, traumatismos do punho

## Abstract

Este estudo mediu a qualidade de vida e os desfechos clínicos e funcionais de um paciente com fratura-luxação radiocárpica submetido a reconstrução aguda dos ligamentos radioescafocapitato (REC) e radiosemilunar (RSL) usando o tendão braquiorradial. O paciente era um homem de 21 anos com fratura-luxação radiocárpica no punho esquerdo após acidente de moto. A fixação percutânea foi realizada com parafuso no rádio distal e reconstrução aguda assistida por artroscopia do REC e RSL. Um ano depois, o paciente apresentou boa avaliação. A amplitude de movimento era completa e a força de preensão era de 96% em comparação ao lado não acometido. Os aspectos radiográficos do punho mostraram a fratura consolidada e congruência da articulação radiocárpica. A boa estabilidade e congruência da articulação radiocárpica melhoraram a qualidade de vida do paciente. O tratamento da fratura-luxação radiocárpica é difícil e complicado. Não há consenso. Como ainda não há resultados em longo prazo, as indicações para cirurgia e opções terapêutica têm sido motivo de controvérsia na literatura. A articulação radiocárpica (RC) seria estável após a reconstrução dos ligamentos radiocárpicos usando o tendão braquiorradial? Seria possível reduzir e manter a articulação radiocárpica estável com esta técnica? A relevância clínica deste trabalho é a nossa sugestão de reconstrução do ligamento RC para melhorar este tratamento. Acreditamos que manteremos o punho estável e funcional. Concordamos que o melhor momento para realizar as correções é o mais breve possível e preferimos reconstruir o ligamento RC com sutura ou artrodese radioescafosemilunar temporária ou definitiva.

## Introdução

A fratura-luxação radiocárpica é um distúrbio traumático incomum associado a lesões nos ligamentos radiocárpico (RC) e radioulnar (RU). O diagnóstico é tardio devido à ausência de achados radiográficos e é feito após falha crônica (instabilidade) da articulação e dor no punho. O tratamento da luxação aguda geralmente requer estabilização e sutura do ligamento. Neste paciente, estabilizamos a articulação radiocárpica por reconstrução do RC usando um enxerto de tendão braquiorradial.

## Descrição do Caso

Um paciente de 21 anos, do sexo masculino, apresentava politraumatismo após um acidente com motocicleta, com dor e deformidade no antebraço dominante esquerdo. As primeiras radiografias iniciais revelaram incongruência da articulação radiocárpica e da articulação radioulnar distal (ARUD) e fratura do estiloide radial ( Fig. 1 ). O paciente foi submetido a tratamento com redução fechada e fixação percutânea (parafuso de compressão sem cabeça HCS, Synthes, Davos, Suíça) no estiloide radial. A seguir, o exame físico revelou RC/ARUD instáveis e uma proeminência dorsal na cabeça ulnar. No intraoperatório, decidimos realizar uma artroscopia do punho e diagnosticamos uma lesão complexa com acometimento dos ligamentos radiocárpicos e avulsão foveal do complexo de fibrocartilagem triangular (CFCT) (os testes de gancho e trampolim foram positivos). Então, abordamos a instabilidade radiocárpica por meio da reconstrução dos ligamentos radiocárpicos (REC, RLL) usando o tendão braquiorradial (BR).

**Fig. 1:** Aspecto radiográfico pré-operatório do punho – fratura-luxação radiocárpica.

O paciente utilizou uma tala longa removível até acima do cotovelo por duas semanas após a cirurgia, com o punho em posição neutra para melhorar a recuperação natural da pronação e supinação. Iniciamos a terapia ocupacional para melhora da amplitude do antebraço na primeira semana após a cirurgia. Quatro meses depois, o paciente estava sem dor e apresentava estabilidade da articulação radiocárpica e ARUD, além de boa amplitude de movimento (ADM) do punho, antebraço, cotovelo e todos os dedos. A pontuação *Disability of Arm, Shoulder and Hand* (DASH) era 6, a escala visual análoga (EVA) de dor era 0 e a força de preensão era de 96% em comparação com o punho contralateral. As imagens radiográficas revelaram a congruência articular da ARUD, a cicatrização do estiloide radial e melhor fixação óssea aos implantes ( Vídeo 1 ).

## Técnica Cirúrgica

Por meio da abordagem radial do punho, identificamos e coletamos o tendão BR, que foi dividido em duas partes: uma para o REC e outra para o RSL. Preparamos o tendão com um suporte interno (fio de sutura FiberTape, Arthrex Inc., Naples, FL, EUA), seccionando-o em sua transição muscular e preservando a inserção no estiloide radial. Depois, empregamos uma abordagem ulnar medial para a reinserção do CFCT na ulna usando a âncora DX por meio de túnel transverso, de acordo com a técnica recomendada pelo fabricante. Depois, criamos três túneis (escafoide, semilunar e capitato) usando uma broca canulada de 3,5 mm de acordo com a técnica descrita pelos autores e auxiliados por artroscopia. Passamos o enxerto do tendão BR através do túnel e o mantivemos tensionado na face palmar no primeiro semilunar ( Fig. 2 ), no escafoide e no capitato ( Fig. 3 ). Assim, estabilizamos a articulação radiocárpica. Os implantes definitivos no escafoide, capitato e semilunar foram inseridos somente após a obtenção da estabilidade (DX). ( Figs. 4 e 5 e Video 1 ).

**Vídeo 1** Caso clínico: aspectos pré, intra e pós-operatórios do diagnóstico ao retorno às atividades diárias.


**Fig. 2:** Desenho esquemático: reconstrução do ligamento radiosemilunar.

**Fig. 3:** Desenho esquemático: reconstrução do ligamento radioescafocapitato.

**Fig. 4:** Aspectos pós-operatórios da radiografia de punho em incidência anteroposterior.

**Fig. 5:** Aspectos pós-operatórios da radiografia do punho em incidência em perfil.

## Discussão

O tratamento convencional sugere redução aberta e fixação do estiloide radial (com parafuso, fios de Kirschner ou placa específica) e reparo direto por sutura dos ligamentos radiocárpico e CFCT com fios de Kirschner. 1

Nas lesões agudas, é possível reparar diretamente os ligamentos radiocárpicos e o CFCT, mas não há consenso sobre o manejo geral.

Nas lesões crônicas (as mais comuns), muitas vezes precisamos reduzir RC/ARUD. Além disso, há necessidade de procedimentos como artrodese parcial do punho, encurtamento da ulna e reconstrução dos ligamentos radiocárpicos. Aita et al. 2 publicaram a reconstrução do REC com resultados promissores na estabilização do punho e prevenção da osteoartrite.

Aita e Mantovani 3 sugeriram um algoritmo para tratamento de lesões ligamentares e introduziram a artroscopia do punho e o Internal Brace para reparo/reconstrução de ligamentos carpais intrínsecos/extrínsecos do punho. A vantagem deste procedimento é a visualização direta das estruturas articulares, evitando a incisão da cápsula dorsal do punho para preservar a propriocepção do membro superior e poupando outros sítios cirúrgicos.

Potter et al. 4 descreveram o “spanning” como um estabilizador da articulação radiocárpica em direção oposta à que tratamos aqui, usando uma placa radiocárpica específica com artrodese temporária e obtenção de resultados positivos.

Aqui, sugerimos que Internal Brace também é suficiente para tratar instabilidades traumáticas de RC e ARUD, pois este é um reparo/reconstrução anatômica semelhante à reconstrução do REC/RSL ou CFCT. Todos os procedimentos preservam a estabilidade e a mobilidade do punho e aumentam a força da reconstrução e sua proteção. 3

Os autores têm ampla experiência no uso do enxerto de tendão BR e oferecem uma solução promissora e eficiente neste caso que parece ser reproduzível em futuras intervenções cirúrgicas. 5

Concordamos que o desfecho em longo prazo, especialmente em um paciente jovem como este, é incerto, e haverá algumas complicações conhecidas e desconhecidas no futuro. Pode haver necessidade de outro procedimento de resgate; no entanto, o excelente resultado funcional neste acompanhamento de médio prazo parece justificar tal indicação nestas circunstâncias especiais.
